# Interaction of Caveolin-3 and HCN is involved in the pathogenesis of diabetic cystopathy

**DOI:** 10.1038/srep24844

**Published:** 2016-04-28

**Authors:** Xingyou Dong, Qixiang Song, Jingzhen Zhu, Jiang Zhao, Qian Liu, Teng Zhang, Zhou Long, Jia Li, Chao Wu, Qingqing Wang, Xiaoyan Hu, Margot Damaser, Longkun Li

**Affiliations:** 1 Department of Urology, Second Affiliated Hospital, Third Military Medical University, Chongqing, China; 2 Department of Biomedical Engineering, the Cleveland Clinic, Cleveland, OH, United States of America

## Abstract

A growing body of research suggests that impaired bladder Cajal-like interstitial cells (ICCs) are a important component in the pathogenesis of diabetes-induced bladder dysfunction, although the molecular mechanisms have not been illustrated completely. The purpose of this study was to examine whether the hyperpolarization-activated cyclic nucleotide-gated (HCN) channels in ICCs-DM were responsible for the detrusor weak contractility of Diabetic cystopathy (DCP) and to study the possible mechanism of regulating the expression and function of HCN channels. HCN channels expression were decreased at the mRNA and protein levels. Forskolin (FSK), which can elevate intracellular cAMP levels, increased the density of the hyperpolarization-activated current and intracellular calcium concentration in both normal control (NC) rats and DCP rats, but the sensitivity of FSK on HCN channels was clearly down-regulated in DCP rats. The loss of caveolae and caveolin was in accordance with the decrease in HCN channels. Caveolin-3 co-localizes with and affects the expression and function of HCN. Taken together, these results indicate that the loss of caveolae and HCN channels in ICCs-DM is important in the pathogenesis of DCP. Increasing the number of caveolae to enhance the function of HCN channels may represent a viable target for the pharmacological treatment of DCP.

Diabetic cystopathy (DCP) is one of the most important complications of diabetes and is characterized by a broad spectrum of lower urinary tract symptoms (LUTS), encompassing urinary urgency, frequency, and incontinence, which are primarily attributable to alterations in neuronal and urothelial detrusor dysfunction^1^. LUTS complications are observed in more than 80% of diabetic patients, a higher rate than that of neuropathy and nephropathy, which have less than 60% and 50%, respectively^2^. DCP is not life-threatening, but it does affect quality of life. Thus, a more comprehensive understanding of how diabetes impacts the genitourinary tract function is imperative.

Diabetes impacts bladder function via two main phases with different mechanisms in a time-dependent progression. In the early phase, hyperglycemia causes compensatory osmotic polyuria-associated myogenic and neurogenic alterations. In the later phase, damaged tissues induce irreversible bladder dysfunction. Diabetic patients commonly fail to recognize urological symptoms sufficiently early; thus, treatment strategies are more likely to be applied at a later stage^3^. The traditional view is that autonomic neuropathy is the only pathophysiological cause of terminal DCP, however, an increasing number of studies have found that myogenic changes may be the direct cause that leads to weak detrusor contractility^4^. The decreased detrusor contractility was associated with the apoptosis of detrusor myocytes^5^ and the distinct deficit of a major group of contraction-related molecules^6^,^7^,^8^. To date, although numerous studies have focused on the myogenic pathogenesis of diabetic cystopathy, the underlying mechanism has not been completely elucidated, and targeted treatment methods are limited.

The Cajal-like interstitial cells (ICCs) were first discovered and were demonstrated to be involved in generating slow-wave activity, driving peristalsis and processing sensory signals in the gastrointestinal tract^9^. Smet *et al.* provided the first evidence that the bladder might contain ICCs by observing cyclic guanosine monophosphate-immunopositive cells in guinea-pig and human bladders, which had a morphological resemblance to gut ICCs^10^. Then ICCs have been identified in tissue sections and whole-mount tissue sheets using antibodies to the inter-mediate filament, vimentin, which is expressed by a broad spectrum of cells of mesenchymal origin, including fibroblasts, myofibroblasts and ICCs. And the c-kit positive ICCs appear to be a subgroup of the larger vimentin positive population. Many groups have adopted the use of the term “ICC” as bladder ICCs could be labelled with tyrosine-protein kinase Kit (c-kit) and were morphologically and ultrastructurally similar to gut ICCs. The functional significance of ICCs in the bladder has not been completely elucidated. However, our group and others have carried out a number of studies to investigate their presence, distribution and functional role in the urinary bladder^11^. A growing number of researchers have argued that ICCs may function as a sensing network, receiving/sending signals from/to the urothelium and detrusor, modulating afferent bladder innervation or activating a spinal or intramural reflex, rather than acting directly as bladder pacemakers^12^.

Histological studies have shown that ICCs are located in the suburothelial region, at the edge of detrusor smooth muscle bundles, and adjacent to sensory nerve endings^13^. The lamina propria ICCs (ICCs-LP) have a stellate-shaped morphology with several branches and are located close to nerves. However, the detrusor muscularis ICCs (ICCs-DM) has two subtypes; elongated cells with several lateral branches and stellate cells similar to ICCs-LP, which are not networked to each other but are arranged in parallel lines in detrusor smooth muscle bundles. Consequently, the different shapes and arrangements indicate that two groups of ICCs have different roles in the physiological function of the bladder^14^. Although both groups of ICCs exhibit spontaneous electrical and Ca^2+^-signaling and respond to application of neurotransmitter substances including adenosine triphosphate and carbachol, differences in the ion channels expressed in bladder ICC subtypes have been demonstrated and this may be indicative of their distinctive functions. The ICCs-LP were considered to be primarily involved in the regulation of sensory processes, while ICCs-DM are involved in the modulation of detrusor contractility. Contraction-related neuroreceptors, such as muscarinic acetylcholine receptors and purinergic receptors, have also been identified in ICCs-DM and are directly involved in the regulation of bladder excitation and contractibility^15^,^16^.

In a recent study, we confirmed that the hyperpolarization-activated cyclic nucleotide-gated (HCN) channel, which was first identified in the heart in the 1980s, was believed to conduct an inward current (Ih) to participate in autorhythmicity and excitability, and was also identified in the rat bladder ICCs-DM, and the HCN channel blocker, ZD7288, can significantly inhibit spontaneous phasic contraction in isolated detrusor strips^17^. This indicates that HCN channels may be a key modulator of ICC function. *Bahareh Vahabi et al.* have reported that ICCs play a role in mediating muscarinic receptor-induced phasic contractions in diabetic rat bladders. The diabetic tissues showed a trend toward reduced sensitivity to the inhibitory effect of the c-kit antagonist imatinib on phasic contractions^18^. Likewise, our group also found a significant decrease in the levels of stem cell factor (SCF), c-kit expression and the number of ICCs-DM in diabetic rat bladders. These results suggest that the alterations in ICCs-DM number and function may contribute to detrusor contractility dysfunction in later phase diabetic bladders^19^.

To the best of our knowledge, it remains unknown whether ICC HCN channels are involved in the occurrence and functional regulation of diabetic bladders. In this study, the HCN channel expression changes, function alteration and their possible mechanisms were investigated using a streptozocin (STZ)-induced type 2 diabetes rat model.

## Results

### Characteristics of DCP rat model

**Figure 1:**
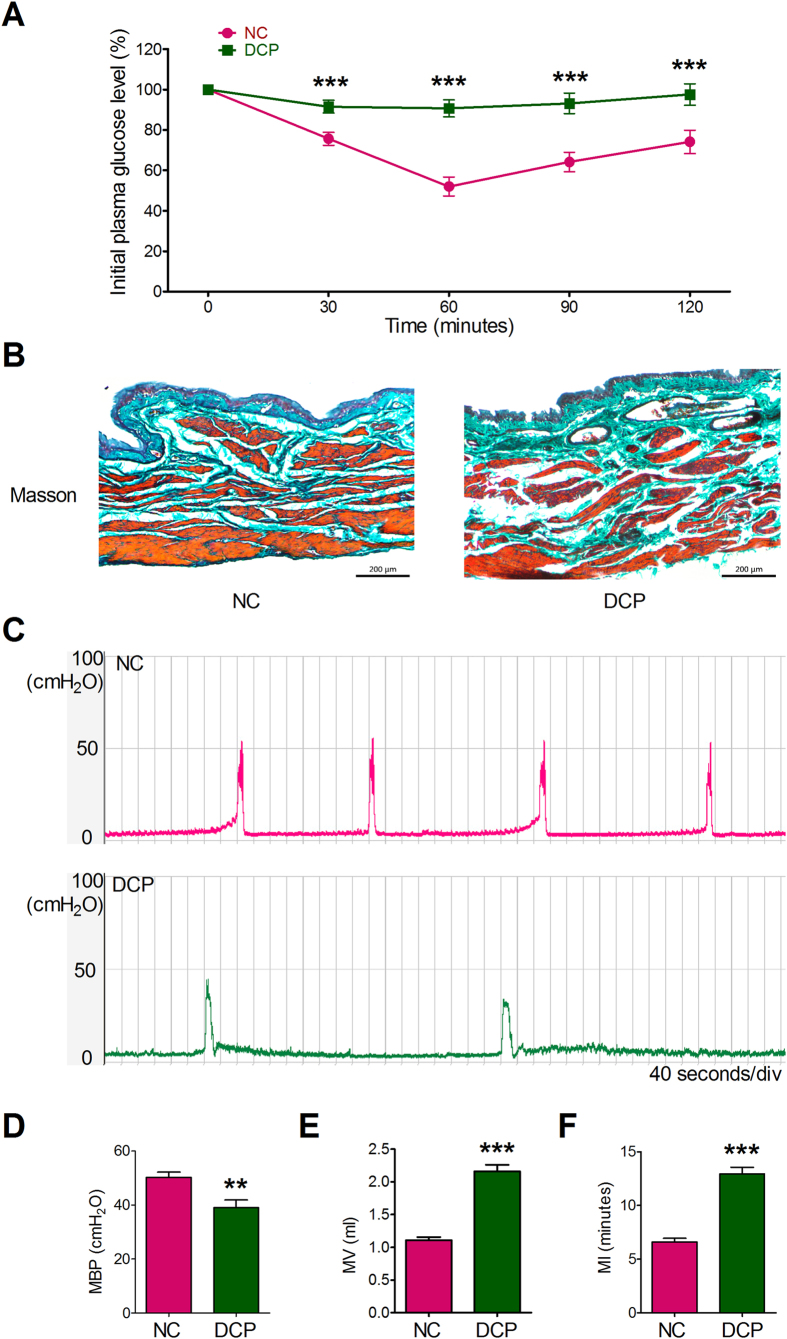
Metabolic and urodynamic characterization of STZ-induced DCP rats. (**A**) Insulin tolerance test was performed 12 weeks after STZ injection. Compared to NC rats, DCP rats lacked a response to insulin (n = 6). (**B**) Increased collagen deposition was detected in DCP rat bladder. (**C**) Typical NC and DCP rat bladder cystometrogram recordings. (**D**–**F**) Effects of diabetes on cystometric parameters. The DCP bladder showed a significant decrease in MBP and increase in MV and MI (n = 30, independent-sample t-test, **P < 0.01 and ***P < 0.001).

Twelve weeks after administration with STZ, the body weight, bladder weight, bladder weight/body weight and fasting blood glucose (FBG) level of type 2 diabetic rats was significantly higher than that of age-matched normal control rats. The food intake, water intake and urine volume in 24 hours were increased in diabetic rats (Table 1). In the insulin tolerance tests (ITT), diabetic rats lacked a response to insulin at 30, 60, 90 and 120 minutes after intraperitoneal injection with insulin (Fig. 1A). The cystometry results showed that the diabetic rats had a decreased maximum bladder pressure (MBP) (Fig. 1D, 50.25 ± 1.89 vs 38.99 ± 2.84 cm H_2_O, P < 0.01) and increased micturition volume (MV, 1.11 ± 0.05 vs 2.16 ± 1.10 ml, P < 0.001) and micturition interval (MI, 6.60 ± 0.37 vs 12.96 ± 0.60 minute, P < 0.001). The diabetic rat bladders showed a decreased bladder voiding efficiency. Masson staining revealed that the thickness of bladder and collagen deposition was increased in DCP rat model (Fig. 1B).

### The decline of HCN channel transcripts and protein in rat diabetic bladder

**Figure 2:**
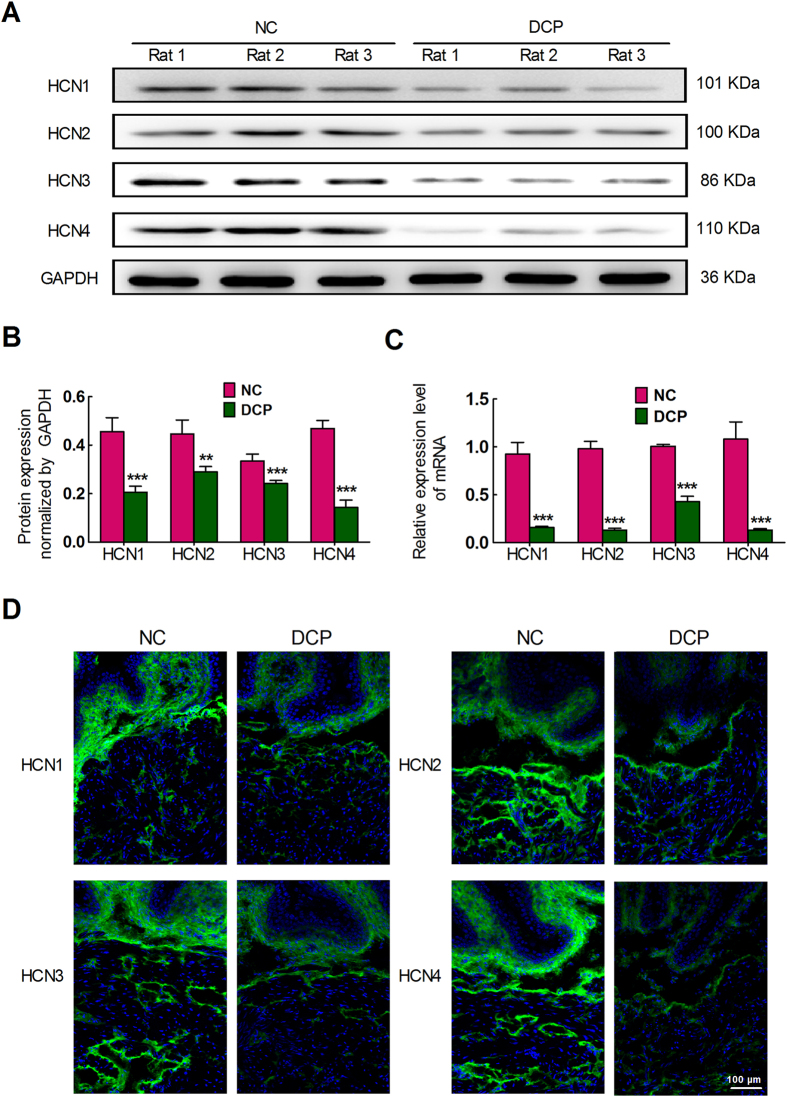
Decreased expression levels of HCN mRNA and protein in DCP bladder ICCs-DM. Western blotting and qRT-PCR showed that diabetes decreased the protein (**A**,**B**) and mRNA (**C**) expression levels of four HCN channels (n = 6, independent-sample t-test, **P < 0.01 and ***P < 0.001). (**D**) Immunofluorescence labeling of HCN channels in rat bladders. Compared to NC rats, the fluorescent density of the four HCN channels was decreased in both the lamina propria and detrusor muscularis of diabetic bladders.

Using real time-quantitative polymerase chain reaction (RT-qPCR) and western blot analysis, we detected the expression alteration of four HCN subtypes in the NC and DCP rat bladder ICCs-DM. The expression of HCN in mRNA and protein was significantly reduced in the ICCs-DM of DCP bladders (Fig. 2A–C). We also analyzed the expression and distribution alteration of HCN using the immunofluorescence method, and the results revealed that all four HCN channel subtypes were mainly distributed in the lamina propria and detrusor muscularis and had a significant decreased protein expression level (Fig. 2D).

### Changes in the properties of Ih currents in ICCs-DM after DCP

**Figure 3:**
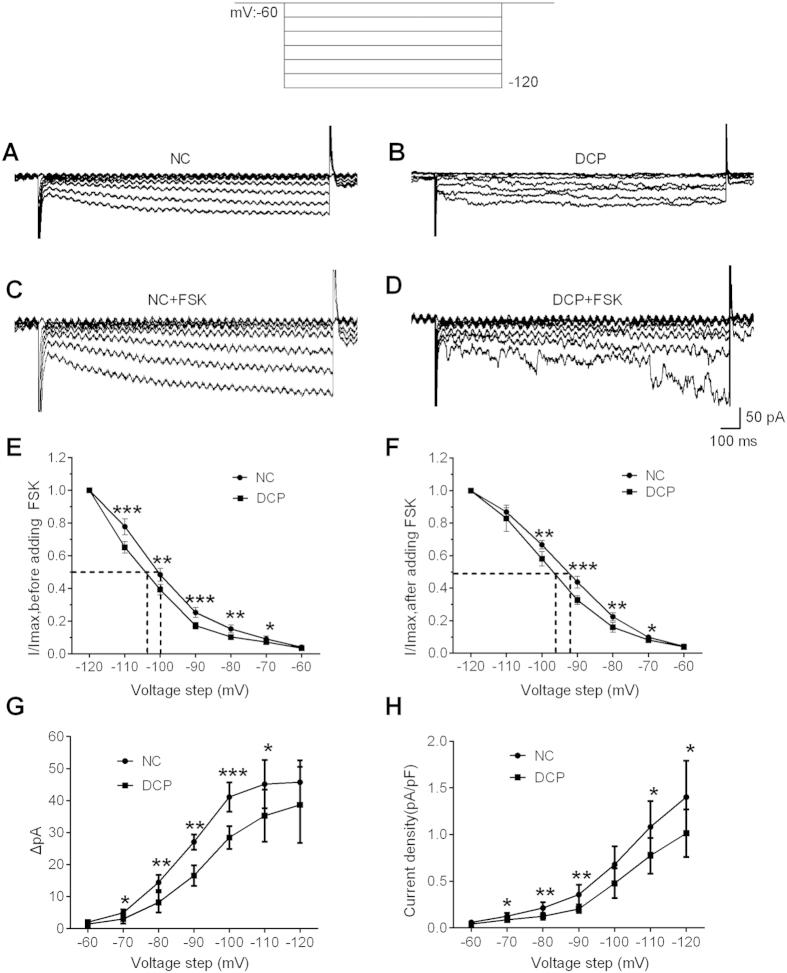
Changes of hyperpolarization-activated Ih currents and FSK sensitivity in DCP bladder ICCs-DM. (**A**,**B**) Representative current traces evoked by hyperpolarizing voltage steps from −120 to −60 mV were recorded in primary isolated NC and DCP rat ICCs-DM. (**C**,**D**) The effects of FSK on Ih currents were detected in both NC and DCP rat ICCs-DM. The voltage dependence of activation was derived from tail current analysis in NC and DCP bladder ICCs-DM. Tail current amplitudes were normalized using the current amplitude at −120 mV as the maximum and fitted with the Boltzmann equation. The estimated half-maximum voltage (V_1/2_) was increased in DCP bladder ICCs-DM. (**F**) FSK increased the Ih currents at different voltage steps and V_1/2_. (**G**) At the hyperpolarizing voltage steps from −120 to −60 mV, FSK increased the Ih currents in both NC and DCP rat ICCs-DM, but had lower sensitivity in DCP rat ICCs-DM. (**H**) Plot of Ih current density versus membrane potential; the amplitude of the Ih current was decreased in DCP bladder ICCs-DM (n = 5, independent-sample t-test, *P < 0.05, **P < 0.01 and ***P < 0.001).

Patch clamp technique was performed to examine the effects of diabetes on the properties of Ih. The voltage dependence of current activation was determined using tail current activation curves (Fig. 3A–D). DCP produced significant diminution of the Ih current amplitude evoked by the −60 to −120 mV voltage-clamp protocol (Fig. 3E). Administered with Forskolin (FSK, 100 μmol), the current amplitudes in both NC and DCP ICCs-DM were increased at different activation potentials (Fig. 3F,G). When normalized to cell capaitance, the Ih current density of DCP ICCs-DM was significantly decreased compared to the Ih current density in NC rats over a voltage range from −90 to −120 mV (Fig. 3H). The membrane potentials of half-maximal activation (V_1/2_) were −100.23 ± 0.45 mV for the NC ICCs-DM and −104.68 ± 0.53 mV for the DCP ICCs-DM. After being treated with FSK, half-maximal activation potentials in both NC and DCP ICCs-DM were increased to −92.87 ± 0.87 mV and −96.47 ± 0.74 mV, respectively. The voltage dependence was shifted to more positive depolarized potentials in ICCs-DM after adding FSK into the bath.

### Enhancement of intracellular calcium concentration induced by FSK

**Figure 4:**
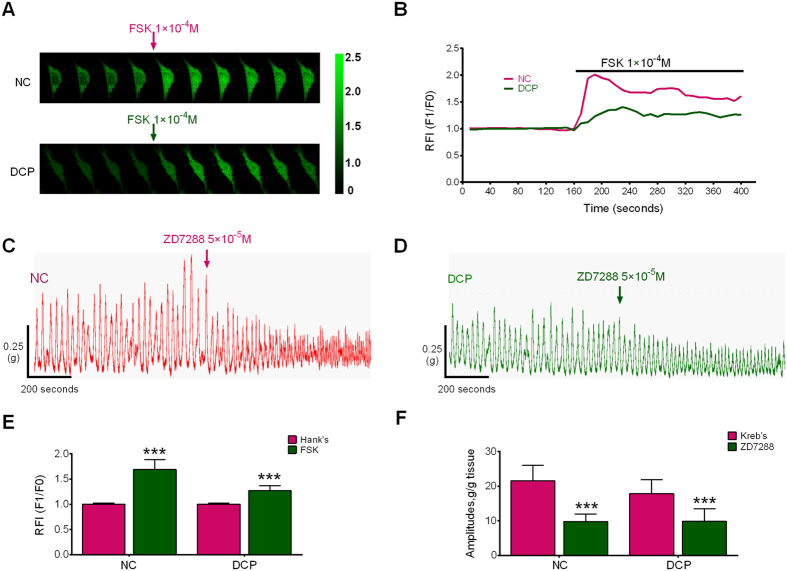
Effects of FSK on the intracellular calcium ion concentration of ICCs-DM. (**A**) Every ten pictures were chosen to represent the process of instantaneous [Ca^2+^]_i_ changes in the ICCs-DM after treatment with FSK. (**B**) The real-time relative fluorescence intensities (RFI, F1/F0) are depicted as continuous plots. FSK at a concentration of 50 μmol/L increases the intracellular calcium concentration in both NC and DCP (**E**) n = 10, independent-sample t-test, ***P < 0.001) rat ICCs-DM, and the increasing effects of FSK on calcium concentration in NC rat ICCs-DM was higher than that in the DCP rat ICCs-DM. (**C**,**D**) Showed the representative detrusor tension recording of NC and DCP rat. ZD7288 decreased the detrusor contraction amplitudes in both NC and DCP rat, but had a higher sensitivity on NC rat detrusor strips (**F**), n = 10, independent-sample t-test, ***P < 0.001).

To detect the effects of FSK on the excitability of bladder ICCs-DM, we measured the intracellular calcium concentration ([Ca^2+^]_i_) using the primary isolated bladder ICCs-DM. FSK increased the intracellular calcium concentration in both NC and DCP bladder ICCs-DM (Fig. 4A,B; RFI: 1.69 ± 0.16 vs 1.00 ± 0.02, P < 0.001), while the DCP bladder ICCs-DM showed a decreased sensitivity to FSK (Fig. 4E,F; RFI: 1.27 ± 0.07 vs 1.00 ± 0.01, P < 0.001).

### Sensitivity of ZD7288 on isolated detrusor strips

To study the effects of ZD7288 on the detrusor contractility, the isolated detrusor strip contractile experiment was performed. ZD7288 can decrease the bladder detrusor contractility in NC and DCP rats (Fig. 4C,D), however the DCP rat detrusor revealed a decreased sensitivity to ZD7288 (Fig. 4G, NC: 21.536 ± 4.458 vs 9.812 ± 2.172 g/g tissue, P < 0.001; Fig. 4H, DCP: 17.832 ± 4.036 vs 9.863 ± 3.584 g/g tissue, P < 0.001).

### Decreased number of caveolae and expression level of caveolin

**Figure 5:**
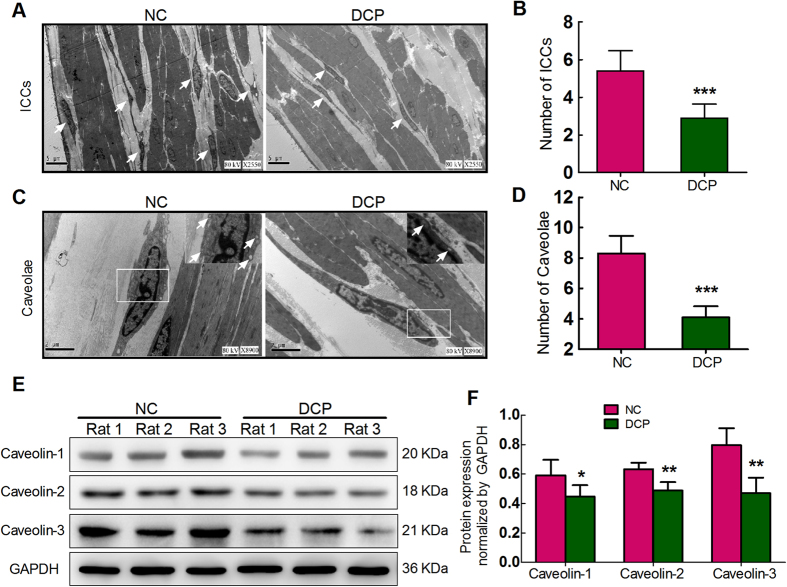
Down-regulation of caveolae number and Caveolin1–3 expressions in DCP bladder ICCs-DM. Electron microscope images of bladders from NC and DCP rats showed the alterations in number of ICCs-DM and density of caveolae (**A**,**C**). Graphs showed ICCs-DM number and caveolae density of DCP bladders decreased (**B**,**D**). (**E**) Western blot for caveolin1–3 in total protein lysate of bladders procured from NC and DCP rats. GAPDH was used as the control. (**F**) Corresponding graph depicts the decreased caveolin protein expression in DCP bladders, normalized by GAPDH (n = 6, independent-sample t-test, *P < 0.05, **P < 0.01 and ***P < 0.001).

The number of ICCs-DM in rat bladders was decreased when the rats underwent significant functional alterations in diabetic state (Fig. 5A,B; 2.90 ± 0.74 vs 5.40 ± 1.08, P < 0.001). To determine whether a diabetes-induced decrease in caveolae is associated with altered caveolin protein expression in ICCs-DM, the number of caveolae and expression of caveolin proteins were determined by transmission electron microscope and western blotting. Fig. 5C,D reveal that the number of caveolae in ICCs-DM was decreased in diabetic bladders (4.10 ± 0.74 vs 8.30 ± 1.16, P < 0.001). The expression levels of three caveolin subtypes were decreased in DCP rat bladder (Fig. 5E,F).

### Co-localization between Caveolin-3 and HCN channels

**Figure 6:**
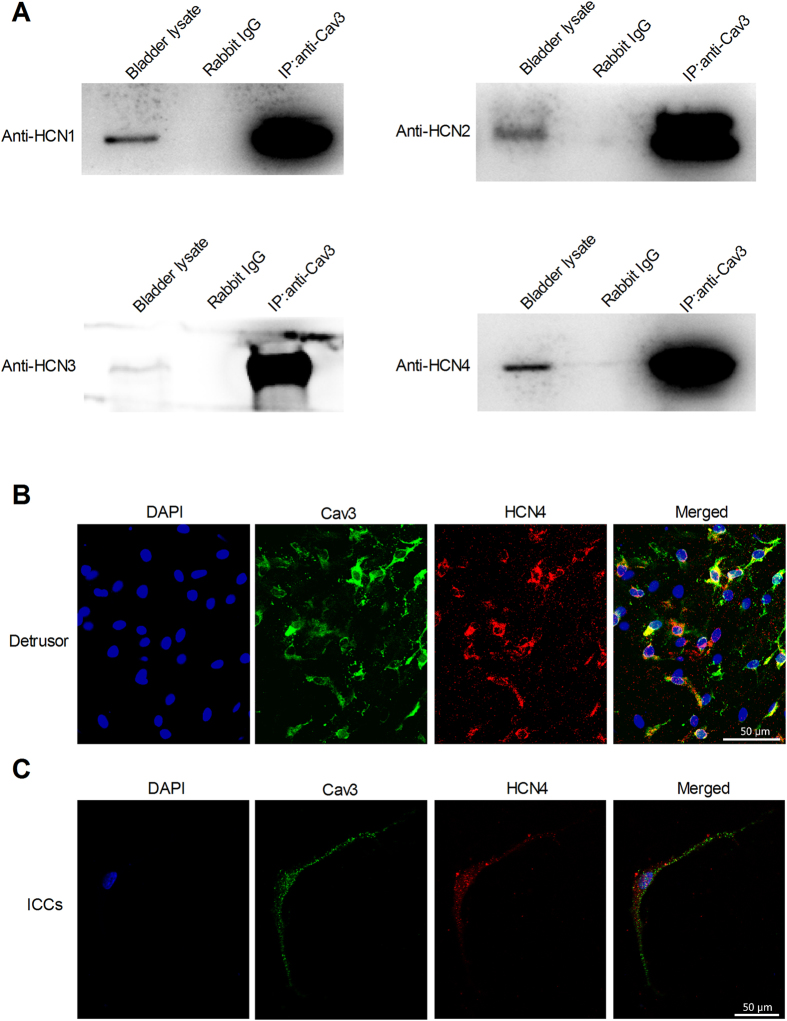
Colocalization and interaction of Cav-3 and HCN channels. (**A**) Representative images from Co-IP analysis of HCN channels and Cav-3. Rat bladder lysate was Co-IPed with anti-Cav-3 antibody or rat IgG, and then the Co-IP samples were analyzed by Western blotting with anti-HCN antibodies (left lane: bladder lysate; mid lane: rabbit Ig G; right lane: Cav-3 Co-IP samples). (**B**,**C**) Confocal images from rat bladder and primary isolated ICCs-DM reveals the co-localization of HCN4 and Cav-3 by immunostaining with rabbit anti-HCN4 polyclonal antibody and rabbit anti-Cav3 polyclonal antibody.

To further study the relationships between caveolin and HCN channels, we performed co-immunoprecipitation (Co-IP) experiments from normal rat bladder lysates that stably expressed Cav-3 protein. The Co-IP samples were analyzed by western blot with anti-HCN1-4 and anti-Cav-3 antibodies. The protein bands for HCN1-4 (101 kDa, 100 kDa, 86 kDa and 110 kDa, respectively) were detected in both bladder lysates and Cav-3 antibody extracted mixed protein, but not in the nonimmune IgG lane (negative control). All four HCN channel proteins appeared to interact with Cav-3 (Fig. 6A). Double immunofluorescent staining of bladder tissue and cultured ICCs-DM revealed that the co-localization of HCN4 and Cav-3 proteins in ICCs-DM can be inferred by merging the green and red channels in the same focal plane, as shown by the yellow marking (Fig. 6B,C).

### Knockdown of Cav-3 inhibited the expression and function of HCN channels

**Figure 7:**
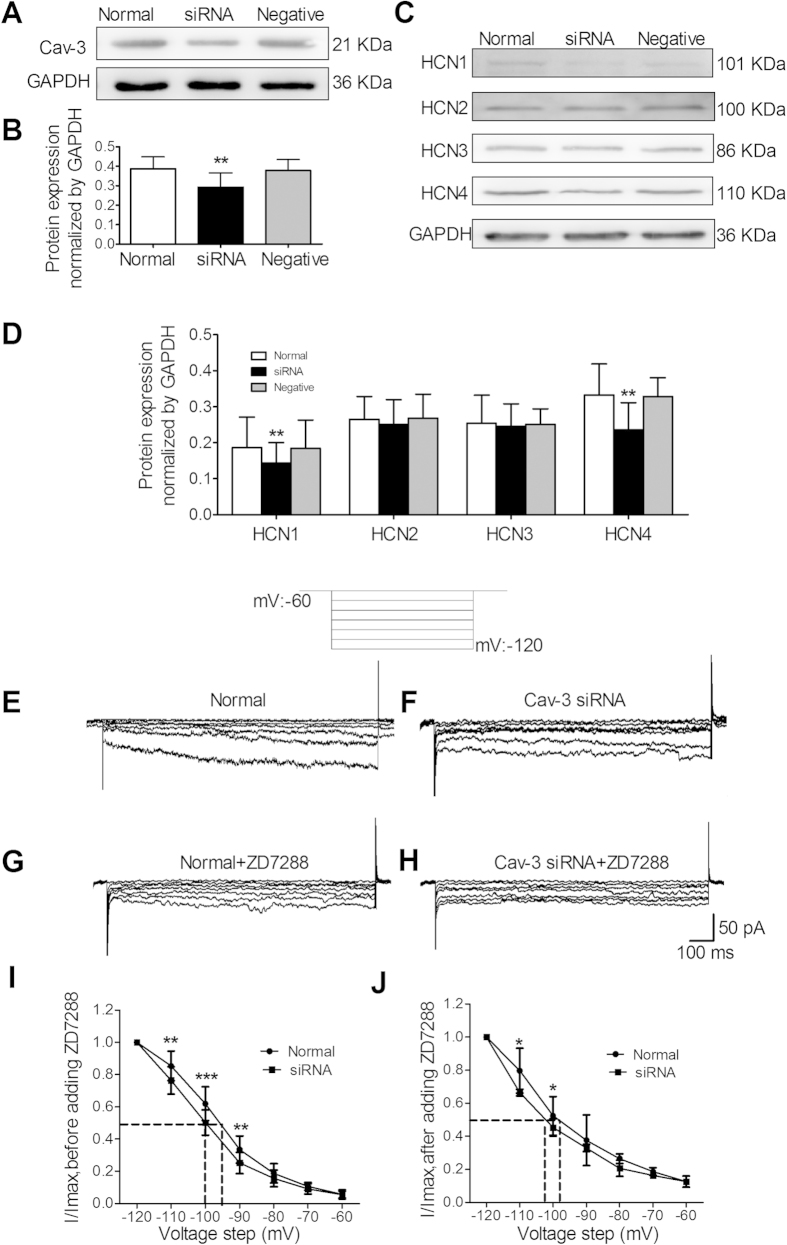
Effects of Cav-3 on the expression and function of HCN channels. Transfection with Cav-3 siRNA in ICCs-DM decreased the protein and function levels of HCN channels. (**A**,**B**) The decreased expression level of Cav-3 was observed only in Cav-3 siRNA transfecting ICCs-DM. (**C**,**D**) Transfection with Cav-3 siRNA can significantly inhibit the protein expression level of HCN1 and HCN4. (**E**,**F**) Representative current traces evoked by hyperpolarizing voltage steps from −120 to −60 mV were recorded in primary isolated normal and Cav-3 siRNA transfecting ICCs-DM. (**G**,**H**) Administration with ZD7288 inhibited the Ih currents in both normal and Cav-3 siRNA transfecting ICCs-DM. Transfection with Cav-3 siRNA decreased the V_1/2_ and Ih currents at the potential of −110, −100 and −90 mV. (**J**) The Ih currents at −110, −100 mV were also decreased by adding ZD7255 (n = 4, independent-sample t-test, **P < 0.01 and ***P < 0.001).

To detect the regulation of Cav-3 on HCN channels, the small interference RNA (siRNA) was added into the culture medium. Figure 7A shows an immunoblot probed for Cav-3 protein in cell lysates harvested after 72 h transfection with Cav-3 specific siRNA. Cav-3 expression was efficiently knocked down by transfection with Cav-3 siRNA, however, transfection with a control random sequence siRNA (con siRNA) did not affect the expression of Cav-3 (Fig. 7B). siRNA-mediated knockdown of Cav-3 protein significantly decreased HCN1 and HCN4 expression. However, no significant differences were observed in the expression of HCN2 and HCN3 (Fig. 7C,D). Accompanying with the decreasing of HCN protein levels, the HCN currents were inhibited at different activation potentials (Fig. 7E–H). The Ih current density of Cav-3 siRNA transfecting ICCs-DM was significantly decreased compared to the Ih current density in normal ICCs-DM over a voltage range from −90 to −120 mV. The voltage dependence was shifted to more negative depolarized potentials (Fig. 7I; V_1/2_: −94.25 ± 2.96 vs 99.50 ± 2.21 mV) Administration with ZD7288 (50 μM) decreased the half-maximal activation potentials in both normal and Cav-3 siRNA transfecting ICCs-DM (Fig. 7J; −97.85 ± 2.54 vs −102.36 ± 2.18 mV).

## Discussion

Our previous work demonstrated that changes in ICCs might be involved in the development of DCP, but the underlying mechanism has not been elucidated^19^. In the present study, the major results reveal that the development of DCP was accompanied by significant decreases in the expression and function levels of HCN channels. This finding may indicate that HCN channels play an important role in DCP.

HCN channels belong to the superfamily of voltage-gated pore loop channels with four pore-forming subunits (HCN1-4) encoded by the HCN1-4 gene, and are activated upon hyperpolarization of membrane potential and conduct an inward, excitatory current. Although Ih shows the same characteristics, such as activation at hyperpolarized membrane potentials, permeability to Na^+^ and K^+^, modulation by intracellular cyclic adenosine monophosphate (cAMP) and blockage by extracellular Cs^+^, many studies have shown that the expression of four HCN subtypes varies among different cells and disease states^20^.

In the present study, the rat DCP models were induced by being fed high-fat diets, injected intraperitoneally with twice low doses of STZ, and identified by FBG, insulin tolerance tests and cystometry at 12 weeks post-injection. The HFD and STZ-induced diabetic rats were characterized by high blood glucose, impaired glucose tolerance, and insulin resistance. In the later phase of DCP, the bladder weights, water intake, food intake were significantly increased. A collagen deposition and smooth muscle thickening also revealed decompensated augment. The changes in cystometric parameters were accompanied by decreased mRNA and protein expression levels of HCN channels. Using RT-qPCR and western blot techniques, we found that rat bladders express the mRNA and protein of four HCN sutypes, and diabetes decreased all four subtypes expression level in different degree. This finding indicates that the down-regulation of HCN channels may be responsible for the development of DCP.

Our previous study highlighted the expression and function of ICCs-DM in the bladder, the spontaneous phasic contraction can be significantly inhibited by HCN channel inhibitor, ZD7288. HCN channels in ICCs-DM may be involved in the regulation of bladder contractility and excitability. The immunofluorescence staining results showed that the immunoreactivity of four HCN subtypes in both of ICCs-LP and ICCs-DM in DCP bladders were smaller than those in NC bladders. This suggested us that decreased detrusor contractility may be related to lower expression levels of HCN in the detrusor muscularis.

In the patch clamp experiment, we investigated the alteration of the HCN current in the ICCs-DM of diabetic bladders. Compared to normal rats, the HCN current intensity was significantly decreased in diabetic bladder ICCs-DM in a voltage-dependent manner. We also used FSK, which elevates cAMP by directly activating adenylate cyclase, to study the effects of cAMP on HCN currents in normal and diabetic bladder ICCs-DM. In the six transmembrane helices of HCN protein, the C-linker and cyclic nucleotide-binding domain can be referred to as the “cAMP-sensing domain” because they are of functional importance for the cAMP-induced positive shift of the voltage-dependent activation of HCN channels^20^. The activation of HCN can result in a small rise in [Ca^2+^]_i_ and induce Ih current to interact with voltage-dependent calcium channels which could mediate the calcium influx^21^. The results indicated a decreased sensitivity of HCN channels on cAMP at different voltage activations. This further illustrates that not only the expression of HCN channels but also the Ih currents, are decreased to participate in the pathophysiology of diabetic bladders. Urinary bladder detrusor muscle possesses the ability to produce spontaneous action potential and initiate spontaneous contraction. The ability may be partly dependent on the activation of ICCs-DM HCN channels^22^. We also detected the effects of FSK on intracellular calcium ion concentration in NC and DCP rats. FSK can increase the intracellular calcium ion concentration in both NC and DCP rats, while the sensitivity of FSK in diabetic bladders was significantly decreased. The acceleration of intracellular calcium ion activity by FSK could be attributable to activation of the HCN channels. In the isolated detrusor strip contractile experiment, HCN blocker, ZD7288 revealed a lower sensitivity on the contractility of DCP rat. It may help us to understand the role of HCN channels in the spontaneous contraction.

It is known that caveolae, as one type of lipid raft in cell plasma membrane, provide the structural framework for macromolecular signaling complexes and are believed to coordinate multiple cellular processes, including second messenger signaling, recycling of proteins to the membrane, and biophysical properties of ion channels. Caveolae include three structural proteins, Cav-1, Cav-2 and Cav-3. Cav-3 is essential for the formation and maintenance of a substantial number of caveolae^23^.

In urinary bladder, *Rasmussen et al.* proved for the first time that caveolae not only distribute widely in smooth muscle cells but also express in ICCs-DM in urinary bladders^24^. The isoform-specific regional distributions of caveolin proteins in the bladder dome, body and base may contribute to the contractile heterogeneity and facilitate differential modulation of responses to local stimuli^25^. *Samar K et al.* found that caveolae have a significant age-dependent decline in bladder; a significant decline in the expression of Cav-2 and Cav-3 proteins parallels caveolae depletion and is consistent with urodynamic alteration in older animals^26^. An experiment in Cav-1-deficient mice showed that the lack of Cav-1 caused a general impairment of detrusor contractility that is partly compensated for by up-regulation of M_3_ receptors and impaired cholinergic neuroeffector transmission^27^. Evidence has been accumulated indicating that the caveolae plays an essential role in the development of urinary bladder diseases^28^. *Polyak*’s research has shown that the decreased number of caveolae and caveolin protein expression in hypertrophied bladders may contribute to alterations in signal transduction pathways^29^, whereas *Shakirova* provided new evidence that rat detrusor hypertrophy due to outflow obstruction led to an increased density of membrane caveolae to promote contraction in response to bradykinin^30^. The decreased caveolae were associated with impaired purinergic signaling in the detrusor^31^. In our transmission electron microscopy experiment, we also found that the density of caveolae and the expression of three structural proteins were down-regulated in the ICCs-DM of diabetic bladders. Caveolin may be directly involved in the regulation of ICCs-DM function.

In the heart, several studies have shown that HCN4 was previously shown to localize to caveolae to form a macromolecular complex, and Cav-3 (but not Cav-1 or Cav-2) was found to be co-localized with HCN4 and has a distinct mechanism for modulation of HCN4 channel properties^32^,^33^. However, the interaction relationship of Cav-3 and HCN in bladder is unknown. Our immunofluorescent results showed that the bladder ICCs-DM have a high degree of co-expression of Cav-3 and HCN4. Co-immunoprecipitation studies have revealed the interaction of Cav-3 and four HCN subtypes in bladder ICCs-DM. Cav-3 could play a role in the modulation of HCN channel properties. To determine the role of caveolae in regulation of HCN channels, we selectively knocked down the expression of Cav-3 protein by transfecting ICCs-DM with Cav-3 siRNA. The decreased protein expression level and Ih current were observed in Cav-3 siRNA transfecting ICCs-DM, but not in normal and negative siRNA transfecting ICCs-DM. These results indicated that the knockdown of Cav-3 may suppress the expression of HCN channels and alter the HCN channel protein to decrease HCN currents in ICCs-DM.

In conclusion, the present study demonstrates decreased expression and function levels of HCN and Caveolin in STZ-induced T2D rat bladders. To the best of our knowledge, this study is the first to demonstrate that HCN channels in ICCs-DM may be regulated by caveolae and may be responsible for the development of diabetic bladders. It is likely that HCN channels and Caveolae in ICCs-DM will present new opportunities to develop novel treatments for adynamia of detrusor muscle in later phrase of DCP.

## Methods

### Animal

All animal handling and experimental protocols were carried out in accordance with the Guide for the Care and Use of Laboratory Animals, and approved by the Research Council and Animal Care and Use Committee of the Third Military Medical University, China (approval no. SYXK20070002). All efforts were made to minimize animal suffering and to reduce the number of animals used. A total of 64 female Sprague-Dawley rats weighting approximately 220–240 g were used in this study and maintained at room temperature (25 ± 2 °C) under a standard 12-h/12-h light-dark cycle. All rats were randomly allocated into two groups: a normal control group (NC rats, n = 32) and an experimental group (DCP rats, n = 32). The type 2 diabetes rat models were induced as previously described^34^. The experimental group rats were fed a high-fat diet (consisted of 22% fat, 48% carbohydrate, and 20% protein with total calorific value 44.3 kJ/kg), while control group rats received a standard diet. After feeding with a high-fat diet for 4 weeks, T2D rats were injected intraperitoneally twice with STZ (Sigma-Aldrich, USA) at a dose of 35 mg/kg body weight with a 1 week internal. After 3 days of STZ injection, the rats with fasting blood glucose FBG > 16.7 mmol/L were confirmed as being the T2D rats.

### Identification of DCP rat model

After 12 weeks of STZ injection, the rat body weight and bladder weight were measured for calculating bladder weight/body weight ratio. The food intake, water intake and urine volume in 24 hours were determinated using metabolic cage method. Masson staining were performed to evaluate the collagen deposition and smooth muscle thickening of bladder in diabetic rats. The FBG level and ITT were measured to identify the diabetic rat models. ITT was performed as follows: insulin (0.75 IU/kg) was administered by intraperitoneal injection and blood samples were collected at 0, 30, 60, 90, and 120 minutes for the measurement of plasma glucose. The value is presented as a percentage of initial plasma glucose level.

### Cystometry Measurement

The NC and DCP rats were anesthetized by an intraperitoneal injection of 20% urethane (1 g/kg). A PE-50 catheter connected to one channel of a three-limb tube was introduced into the bladder through the urethral opening. The other two channels were connected to a pressure transducer linked with a multi-channel signal acquisition system (RM6240C, Chengyi, Chengdu, China) and a microinjection pump (AVI 270, 3 M, Minnesota, USA). The perfusion of normal saline was kept at a constant speed of 10 ml/hour. The following parameters were recorded and calculated: MBP, MV and MI.

### Detrusor Strips Tension Recording

The bladders were freshly resected from NC and DCP rats. A longitudinal incision from the base to dome was made to flatten the bladder. About 3–4 longitudinal strips about 3 × 3 × 8 mm were then cut from the bladder body and transferred to 10 ml organ bath filled with Kreb’s solution (containing as following: 118.7 mM NaCl, 1.2 mM KH2PO4, 4.7 mM KCl, 2.5 mM CaCl2, 12.5 mM NaHCO3, 1.2 mM MgSO4, and 5. 5 mM glucose). The solution was maintained at 37 °C, and bubbled with a mixture of 95% O2 and 5% CO2 and PH value was kept at 7.4. At the start of experiments, the strips were suspended under 1 g tension in the solution by connecting one end of strip to an isometric force transducer (JZJ01, Chengdu Instrument Factory, Chengdu, China) and fixing the other end to hook on the bottom. After 30 minutes equilibration period, a cumulative concentration of HCN blocker, ZD7288 (50 μm) was used to assess the effects on the spontaneous phasic contraction. The contraction amplitudes were recorded and analyzed using a four-channel physiological signal acquisition processing system (RM6240C, Chengdu Instrument Factory, Chengdu, China).

### Quantitative RT-PCR

The expressions of four HCN subtype mRNAs in the bladders of NC rats and diabetic rats were analyzed using real-time quantitative PCR. Total RNA was isolated using TRIzol reagent (Invitrogen, Carlsbad, CA, USA). The extracted RNA was dissolved in diethylpyrocarbonate-treated water. The concentration of RNA was estimated by measuring the absorbance at 260 nm. Complementary DNA was synthesized using a Rever Tra Ace qPCR Kit (Toyobo Co., LTD., Osaka, Japan). The sequences of the primers used were as follows:

HCN1 forward, 5′-AGGCCCCCGTCAGCATGTC-3′; HCN1 reverse, 5′-GGCATGTCAGCTGGTAACTTG-3′; HCN2 forward, 5′-GGACCCCGAAAAGATAAAGAAAAAG-3′; HCN2 reverse, 5′-GCGCTTGCCAGGTCGTAGGTC-3′. HCN3 forward, 5′-GAGCGCATCCACGAGTACTAC-3′; HCN3 reverse, 5′-CCCGGCAGGTGAAGTTAATAATC-3′; HCN4 forward, 5′-CAGGCACCCGTAGGCATGTC-3′; HCN4 reverse, 5′-GCCCTGGTAGCGGTGTTCGTAG-3′.

Quantitative RT-PCR was performed with a LightCycler 480 (Roche, Carlsbad, CA, USA) using SYBR^®^ Green Realtime PCR Master Mix (Toyobo Co., LTD., Osaka, Japan) according to the following cycle settings: (i) initial heating 95 °C for 30 s and (ii) amplification over 40 cycles at 95 °C for 5 s, and 62 °C for 20 s. The amplified product was subjected to SYBR Green I melting curve analysis by ramping the temperature of the reaction samples up from 60 to 95 °C. A single DNA melting profile was observed under these dissociation assay conditions demonstrating amplification of a single unique product. Expression of each gene was normalized to abundance of mRNA for β-actin.

### Western Blot Analysis

The total protein was extracted from rat bladders and isolated ICCs-DM using RIPA lysis buffer (Beyotime, Haimen, China), and the concentration was measured using the Bio-Rad protein assay (Bio-Rad Lab-oratories, Hercules, CA). 50 μg protein was electrophoresed on SDS-PAGE and transferred onto polyvinylidene fluoride membranes (Merk Millipore, Billerica, USA). The membranes were incubated for 2 hours in blocking buffer (5% bovine serum albumin dissolved in Tris-buffered saline solution) to prevent nonspecific binding. Then, the membranes were incubated overnight at 4 °C with anti-HCN1-4 antibodies (1:1000), rabbit anti-Cav1-3 antibodies (1:1000; Cav-1 and 2 bought from GeneTex, California, USA; Cav-3 bought from Abcam, Cambridge, UK) and rabbit anti-glyceraldehyde phosphate dehydrogenase (GPADH) primary antibody (1:1500, Zhongshan, Peking, China). After being washed in tris buffered saline with tween (10 min × 3 times), the membranes were incubated with horseradish peroxidase-conjugated anti-mouse or anti-rabbit IgG (1: 5000, Zhongshan, Peking, China) for 2 h at room temperature. After incubation of the membranes with enhanced chemiluminescence (ECL, Millipore, Billerica, MA), the protein band images were collected and analyzed by Molecular Image, ChemiDoc XRS Image System (Bio-Rad Laboratories). The density value was counted by scanning with ImageLab software. The density of the band was normalized by GAPDH.

### Immunofluorescence Staining

For the frozen section, the resected bladders were fixed in 4% paraformaldehyde solution for 2 hours at 4 °C. After being washed in phosphate buffered saline (PBS, 0.1 M, pH = 7.4), the tissues were cryoprotected by immersion in 30% sucrose overnight at 4 °C. Then, the tissues were cut into 5 μm sections on a freezing microtome. For the avulsed section, the rat bladders were fixed in 4% paraformaldehyde for 12 hours at 4 °C, and the mucosa was carefully removed. The thin sheet samples of the muscle bundles were prepared. All sections were washed in PBS for 10 min and immersed in immunostaining blocking buffer (Beyotime, Haimen, China) for 30 min at room temperature to prevent nonspecific binding sites on the tissue. The sections on the slides were incubated overnight at 4 °C with four HCN primary antibodies (purchased from Abcam, Cambridge, UK): mouse anti-HCN1 (ab84816, 1:200), rabbit anti-HCN2 (ab65704, 1:200), rabbit anti-HCN3 (ab65705, 1:200), rabbit anti-HCN4 (ab32675, 1:200) and rabbit anti-Cav-3 (ab2912, 1:200). After rinsing in Phosphate buffer saline (PBS) (10 min × 3 times), the slides were incubated with fluorescence-conjugated Alexa 488 rabbit anti-mouse and goat anti-rabbit IgG (1:500, Beyotime, Haimen, China) for 1 hour at room temperature. Next, the preparations were washed in PBS (10 min × 3 times) and incubated with 4′;6-diamidino-2-phenylindole (DAPI, Beyotime, Haimen, China) to label the cell nucleus. In each run, negative controls (no primary antibody) were also included. The pictures were captured by laser confocal microscopy. The mean intensity value of fluorescence was calculated using ZEN imaging software (Carl Zeiss AG, Jena, Germany).

### Preparation of Isolated ICCs-DM

Primary ICCs-DM were isolated using a two-step enzymatic digestion method as described previously^35^. After removed the urothelium and lamina propria, the bladder detrusor was chopped into small pieces and incubated in digestion solution A containing 0.2 mg papain (Sigma-Aldrich), 1 mg dithiothreitol (Sigma-Aldrich), and 1 mg BSA (Sigma-Aldrich) for 5 minutes at 37 °C. The tissues were then transferred into digestion solution B containing 1 mg type II collagenase (Sigma-Aldrich), 1 mg dithiothreitol, and 1 mg BSA for an additional 30 to 35 minutes. The cells were cultured in Ca^2+^ free D-hank’s solution to adhere to the bottom of the chamber. Cells with a typical morphology of many branches under visible light were prepared for subsequent experiments as reported by McCloskey^11^.

### Patch Clamp

The ICCs-DM were cultured for two days and then held at −60 mV. Ih currents were recorded using an Axopatch 700-B system (Axon Instruments, Sunnyvale, CA, USA) and an analog-digital converter (Digidata 1200, Axopatch version 9, Axon Instruments) at 4 kHz and filtered at a threshold frequency of 3 kHz. HCN currents were allowed to be partially activated by hyperpolarizing test commands within a range of −60 to −120 mV in −10 mV increments. The cell was then subjected to a voltage jump to −120 mV to obtain full activation of HCN currents. The currents were normalized to cell capacitance. FSK and ZD7288 were used to detect the properties of Ih currents. The electrodes were pulled (P-87; Sutter Instrument,Novato, CA). The extracellular solution contains (in mM): NaCl, 140; MgCl_2_, 1.2; KCl, 5.4; CaCl_2_, 1.8; D-glucose, 10 and HEPES, 5. The pipette solution contained (in mM): K aspartate, 130; Na_2_GTP, 0.1; Na_2_ATP, 5; MgCl_2_, 2; CaCl_2_, 5; EGTA, 11 and HEPES, 10. Data were recorded at room temperature using an Axo multipatch 200B amplifier and analyzed using pCLAMP 10.2 software (Molecular Devices, Sunnyvale, CA).

### Measurement of Intracellular Calcium Ion Concentration

After undergoing adherent culture for 8 hours, the primary isolated ICCs-DM were incubated with Fluo-4 AM (5 μM, Calcium Ion Probes) for 30 minutes at 37 °C. Ca^2+^ imaging experiments were captured at an emission wavelength of 488 nm using laser confocal microscopy. FSK (100 μM) was used to detect the effects in real-time intracellular calcium ion concentration ([Ca^2+^]_i_). The effects are presented as the relative fluorescence intensities (RFI  = F1/F0, where F1 is the real-time fluorescence intensity, and F0 is the baseline fluorescence intensity before administration).

### Transmission Electron Microscope

The bladder tissue samples were freshly harvested and fixed by immersion in 2.5% glutaraldehyde in 0.1 M cacodylate buffer at 4 °C for 4 h and 1% perosmic acid for 2 h. After ethanol gradient dehydration, the specimens were embedded in Epon812 ethoxyline resin at 60 °C for 48 h. The ultrathin sections were cut at 60–80 nm and stained with 1% uranyl acetate. The examination was performed using transmission electron microscope (Philips CM10; Eindhoven, Netherlands) at 80 kV. Low magnification (8900×) and high magnification (15000×) images were obtained to calculate the numbers of ICCs-DM and caveolae.

### Co-Immunoprecipitation

Cav-3, HCN1-4 antibodies, and rabbit IgGs were immobilized on magnetic beads. Antibody coupled beads were incubated with bladder lysates for 1 hour. Immunocoplexes were separated from beads by placing the tubes in a magnetic field. Purified protein complexes were separated by electrophoresis and transferred to membrane. Interaction between Cav-3 and HCN1-4 was evaluated by probing Cav-3 immunoprecipitating HCN1-4 with Cav-3 antibody.

### SiRNA knocks down Caveolin-3

In order to selectively interfere the expression of Cav-3 in ICCs-DM, we designed a SiRNA duplex targeted to the rat Cav-3 (Gene ID: NM_019155.2), sequence sense 5′-GGGCACUUACAGCUUCGAU-3 and antisense 5-AUCGAAGCUGUAAGUGCCC-3, starting at 282 from the open reading frame (136–591) of rat Cav-3 mRNA. The RNA sequence used as a negative control for siRNA activity was: 5-GGGATTCCGACCTTACGAT-3. Small interfering RNA duplex oligonucleotides were purchased from Genepharma (Shanghai, China). The experiments were conducted 72 h after transfection. To assess the specific effect of Cav-3 siRNA on silencing Cav-3 expression, the protein levels of Cav-3 were detected by western blot analysis.

### Statistical Analyses

All experimental data are presented as means ± SD. One-way ANOVA tests performed in SPSS 16.0 software (SPSS Inc., Chicago, IL) were used for the comparisons. Differences with P values below 0.05 were considered significant.

## Additional Information

**How to cite this article**: Dong, X. *et al.* Interaction of Caveolin-3 and HCN is involved in the pathogenesis of diabetic cystopathy. *Sci. Rep.* **6**, 24844; doi: 10.1038/srep24844 (2016).

## Figures and Tables

**Table 1:** Rat metabolic parameters in each group.

Groups	NC	DCP
Body weight (g)	362.59 ± 25.69	329.91 ± 18.56***
Bladder weight (mg)	127.21 ± 11.40	316.47 ± 21.15*
Bladder weight/Body weight (mg/g)	0.35 ± 0.02	0.96 ± 0.05***
Fasting blood glucose (mmol/L)	4.63 ± 0.51	23.97 ± 1.85***
24 h food intake (kj)	66.99 ± 4.67	82.70 ± 5.34***
24 h water intake (ml)	40.91 ± 5.80	139.26 ± 14.60***
24 h urine volume (ml)	11.27 ± 1.15	52.54 ± 4.72***

Data are presented as means ± SD (n = 7). NC = normal control rats; DCP = diabetic cystopathy rat. The body weight, bladder weight, bladder weight/body weight, fasting blood glucose, food intake, water intake, urine volume in 24 hours were increased in DCP rats.

^*^Indicates a significant difference compared to NC group (P < 0.05).

^***^Indicates a significant difference compared to NC group (P < 0.001).
